# Possible Reactions of Dietary Phenolic Compounds with Salivary Nitrite and Thiocyanate in the Stomach

**DOI:** 10.3390/antiox6030053

**Published:** 2017-07-05

**Authors:** Umeo Takahama, Sachiko Hirota

**Affiliations:** Department of Health and Nutrition Care, Faculty of Allied Health Sciences, University of East Asia, Shimonoseki 751-8503, Japan; hirotasa@toua-u.ac.jp

**Keywords:** flavonoids, nitric oxide (•NO), nitrosation, nitrous acid, quinones, redox reactions, stomach, thiocyanic acid

## Abstract

Foods are mixed with saliva in the oral cavity and swallowed. While staying in the stomach, saliva is contentiously provided to mix with the ingested foods. Because a salivary component of nitrite is protonated to produce active nitrous acid at acidic pH, the redox reactions of nitrous acid with phenolic compounds in foods become possible in the stomach. In the reactions, nitrous acid is reduced to nitric oxide (•NO), producing various products from phenolic compounds. In the products, stable hydroxybezoyl benzofuranone derivatives, which are produced from quercetin and its 7-*O*-glucoside, are included. Caffeic acid, chlorogenic acid, and rutin are oxidized to quinones and the quinones can react with thiocyanic acid derived from saliva, producing stable oxathiolone derivatives. 6,8-Dinitrosocatechis are produced from catechins by the redox reaction, and the dinitrocatechins are oxidized further by nitrous acid producing the quinones, which can make charge transfer complexes with the dinitrosocatechin and can react with thiocyanic acid producing the stable thiocyanate conjugates. In this way, various products can be produced by the reactions of salivary nitrite with dietary phenolic compounds, and reactive and toxic quinones formed by the reactions are postulated to be removed in the stomach by thiocyanic acid derived from saliva.

## 1. Introduction

Phenolic compounds are normally contained in terrestrial plants, and their various functions have been reported. Protection of mesophyll cells from UV-light, scavenging of reactive oxygen species, protection from microbial infection, avoiding from feeding damages, and so forth are included among their functions [1,2,3,4]. In humans, phenolic compounds derived from plants also have various functions. The functions can be roughly divided into two categories; one is antioxidative activity and the other is the modulation of functional proteins. The scavenging of superoxide anion radicals and hydroxyl radicals by redox reactions and scavenging of hydrogen peroxide by salivary peroxidase/phenolic compound systems in the oral cavity are included in the former, and binding to hormone receptors and some enzymes regulating their activity are categorized into the latter [4,5,6,7].

Accompanying the ingestion of foods that contain phenolic compounds, the compounds are mixed with saliva, which contains 0.05–1 mM nitrite produced by oral nitrate reducing bacteria [8,9]. Salivary nitrate, which is originated from vegetables, is provided by enterosalivary circulation. Swallowed foods are stay in the stomach for 1–3 h, and are then transported to the intestine. While in the stomach, salivary nitrite is continuously provided to the swallowed foods. In the stomach, nitrite is protonated producing nitrous acid (p*K*_a_ = 3.3), an oxidizing and nitrosating agent, to react with food components. It is well known that nitrous acid can transform amines into carcinogenic compounds nitrosoamines [10], and it has been reported that phenolic compounds can reduce nitrous acid to a functional compound nitric oxide (•NO) [11,12]. The functions of •NO produced in the gastric lumen include the increase in gastric mucosal blood flow, the increase in gastric mucus thickness, and the relaxation of gastric smooth muscle [13,14,15,16]. 

On the other hand, there are many reports that nitrite/nitrate absorbed from the intestine can contribute to the production of •NO in human cells [17,18]. The functions include the lowering blood pressure by vasodilatation [19,20,21], and the control of platelet aggregation and vascular smooth muscle cell proliferation [22,23,24,25]. Recently, nitrite-induced activation of endothelial •NO synthase is reported [26]. 

This review deals with the redox reactions of phenolic compounds with nitrite to produce •NO and the various reactions of the products of phenolic compounds with •NO, nitrite, and a salivary component thiocyanate under the simulated stomach conditions.

## 2. Reactions of Phenolic Compounds with Nitrite

### 2.1. Nitric Oxide (•NO) Formation

It is known that •NO is produced in the stomach [27,28], and its production is enhanced by the administration of nitrite/nitrate [29] and by the ingestion of nitrate-rich leafy vegetable such as spinach and lettuce [8,30]. As a mechanism of •NO production, self-decomposition of nitrous acid (p*K*_a_ = 3.3), which is produced by nitrate-reducing bacteria in the oral cavity [8,9], has been proposed [29,31]

2HNO_2_ → N_2_O_3_ + H_2_O (1)

N_2_O_3_ → •NO + •NO_2_ (2)

Another mechanism of the nitrite-dependent production of •NO is the reduction of nitrous acid by ascorbic acid and phenolic compounds by the following reactions [11,12,32]

HNO_2_ + ascorbic acid → •NO + monodehydroascrobic acid radical (3)

HNO_2_ + phenolic compounds → •NO + H_2_O + radicals of phenolic compounds (4)

Monodehydroascorbic acid radicals produced by reaction 3 are transformed into ascorbic acid and dehydroascorbic acid by disproportionation. Phenolic compounds with a catechol group, such as quercetin, catechins, caffeic acid, chlorogenic acid can reduce nitrous acid to •NO rapidly by reaction 4 [11,12,33]. In contrast to these compounds, kaempferol (a flavonol with a monohydroxyl group in the B ring), rutin (a quercetin 3-rutinoside), and two kinds of flavone (apigenin, and luteolin) are much less reactive with nitrous acid [11], suggesting that both a catechol group in the B ring and a free hydroxyl group in the C ring are required for the rapid reaction. The •NO produced in the stomach can be measured by the increases in •NO concentration in the expelled air from the stomach [28]. The •NO concentration increases after drinking wine [34,35] and after eating dough produced from buckwheat flour, which contains large amounts of phenolic compounds [36]. 

In addition to the reduction of nitrous acid by reaction 3, ascorbic acid can reduce radicals of phenolic compounds. This is deduced from the results that nitrous acid-induced oxidation of quercetin and chlorogenic acid is inhibited by ascorbic acid [37,38] and the inhibition accompanies the formation of monodehydroascorbic acid radical [38]. The ascorbic acid-dependent inhibition of their oxidation suggests that ascorbic acid in gastric juice, the concentration of which ranges from 0 to 0.5 mM (average, about 0.1 mM) [39,40,41], can suppress the nitrous acid-induced oxidation of phenolic compounds in the stomach. These results suggest that efficiency of the transport of phenolic compounds, which can react with nitrous acid readily, to the intestine is dependent on the concentrations of both salivary nitrite and gastric ascorbic acid.

**Figure 1:**
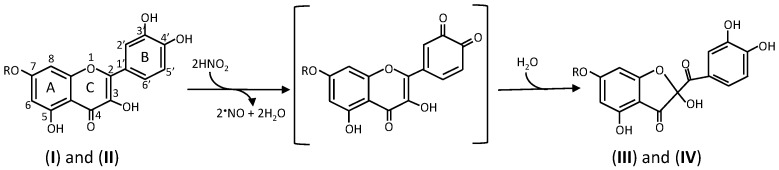
Reactions of quercetin **I** (R = H) and its 7-*O*-glucoside **II** (R = glucose) with nitrous acid. (**III**) 2-(3,4-dihydroxybenzoyl)-2,4,6-trihydroxy-3(*2H*)-benzofuranone, (**IV**) 2-(3,4-dihydroxybenzoyl)-2,4-dihydroxy-3(*2H*)-benzofuranone 6-*O*-glucoside. The compounds in brackets are unstable quinones of **I** and **II**.

When the ascorbic acid concentration is lower than the nitrite concentration in the stomach, oxidation products of phenolic compounds can be produced via radicals generated by reaction 4. Recently, it has been reported that 2-(3,4-dihydroxybenzoyl)-2,4,6-trihydroxy-3(*2H*)-benzofuranone (**III** in Figure 1), which has antioxidative activity [42], is produced when quercetin (**I**) is incubated with nitrous acid [43] and when a food prepared using adzuki bean, which contains quercetin, is incubated with nitrite under the simulated stomach conditions [44] (Figure 1). It is known that in addition to **III**, 2,4,6-trihydroxyphenylglyoxylic acid and 3,4-dihydroxybenzoic acid are included in the stable oxidation products of quercetin [45,46,47,48,49,50]. It is also reported that nitrite-induced oxidation of quercetin 7-*O*-glucoside (**II**) under acidic conditions results in the formation of a glucoside of 2-(3,4-dihydroxybenzoyl)-2,4,6-trihydroxy-3(*2H*)-benzofuranone (**IV**) in addition to •NO [51]. Compounds **III** and **IV** seem to be formed from the unstable *ortho*-quinones of quercetin and quercetin 7-*O*-glucoside, respectively, which are produced by the disproportionation of quercetin and quercetin 7-*O*-glucoside radicals [48,49].

Thus, quinones of quercetin and its glycosides with both a 3-hydroxyl group in the C ring and a quinone structure in the B ring are rapidly transformed into a stable compounds by reacting with water [48] even if they are produced in the stomach. Because compound **III** has antioxidative activity, compound **IV** can also function as antioxidant if hydrolyzed in the intestine. It is known that compound III is present in a large amount in brown outer scales of onion [42].

### 2.2. Formation of Oxathiolone Derivatives

**Figure 2:**
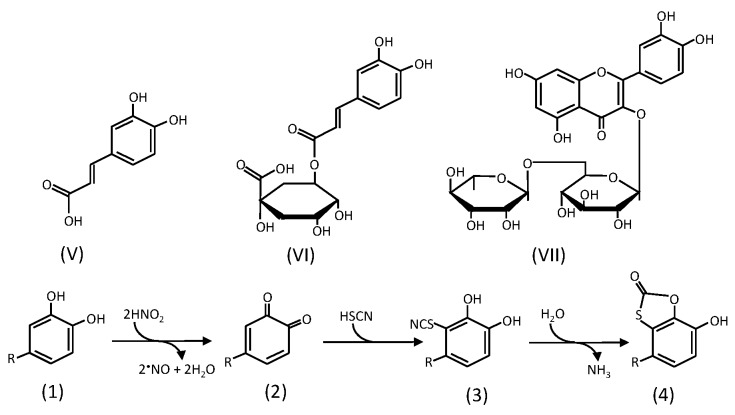
Postulated mechanism of the formation of oxathiolone derivatives from phenolic compounds [33,53]. (**V**) caffeic acid, (**VI**) chlorogenic acid, (**VII**) rutin. (1) phenolic compound with a catechol group, (2) quinone of the phenolic compound, (3) thiocyanate conjugate, (4) oxathiolone derivative.

Although the quinones of quercetin and quercetin 7-*O*-glucoside can react rapidly with water producing the stable oxidation products as mentioned above, *ortho*-quinones, which are stable at acidic pH, are produced from the phenoxyl radicals of caffeic acid (**V**), chlorogenic acid (**VI**), and rutin (**VII**) (Figure 2) [33,52,53]. The quinones are able to react with thiocyanate, which is a salivary component, under acidic conditions producing stable oxathiolone derivatives [33,53]. As the mechanism of their production, reactions in Figure 2 are proposed. At first, the quinones produced by two-electron oxidation react with thiocyanic acid, producing thiocyanate conjugates. Then, the thiocyanate group and the adjacent hydroxyl group react with each other, consuming water and producing ammonia, resulting in the formation of oxathiolones. 

Although the quinones of rutin, caffeic acid, and chlorogenic acid are stable under acidic conditions, they become unstable with the increase in pH to 7 [52]. The unstableness suggests that if the quinones formed in the stomach are absorbed into the body, they can react with other components and can decompose by self-reactions in cells. It has been reported that quinones can react with not only ascorbic acid but also glutathione [54] and amino acids [55], and it is discussed that the quinones can contribute to the production of hydroxyl radicals if hydrogen peroxide reacts with semiquinone radicals formed from quinones and hydroquinones [56]. The self-reactions of quinones may result in the production of polymers like melanins that are produced from the quinone of 3,4-dihydroxyphenylalanine (dopa quinone). Then, the physiological function of the reaction of thiocyanaic acid with *ortho*-quinones can be postulated to the removal or stabilization of quinones generated from the phenolic compounds. 

The formation of an oxathiolone derivative of caffeic acid in a mixture of saliva and acidic white wine suggests that its formation is possible in not only the oral cavity but also the stomach after drinking wine [35]. From this report, we can deduce that the ingestion of beverages and foods rich in phenolic compounds—such as caffeic acid, chlorogenic acid, and rutin—can result in the formation of their oxathiolone derivatives via the quinones in the stomach. The efficiency of the removal of *ortho*-quinones by the reactions in Figure 2 in the stomach is dependent on the concentrations of thiocyanate in saliva. It has been reported that salivary concentration of thiocyanate ranges from 0.01 to 3 mM [57]. 

On the other hand, some pharmacological functions of the oxathiolone derivatives are reported. The functions include antibacterial and antifungal activity toward various microorganisms and cytotoxic activity for HeLa cells [58], suppression of a nuclear factor activation [59], and inhibition of xanthine oxidase activity [60]. An oxathiolone derivative of quercetin, which can inhibit xanthine oxidase activity more effectively than quercetin, is prepared by hydrolysis of an oxathiolone derivative of rutin [60]. Then, if an oxathiolone derivative of rutin is produced in the stomach, the formation of the oxathiolone derivative of quercetin is possible in the intestine. The oxathiolone derivative of quercetin seems not to be easily formed from quercetin because of the rapid reaction of its quinone form with water as described above. 

### 2.3. Reactions of Catechins with Nitrous Acid

**Figure 3:**
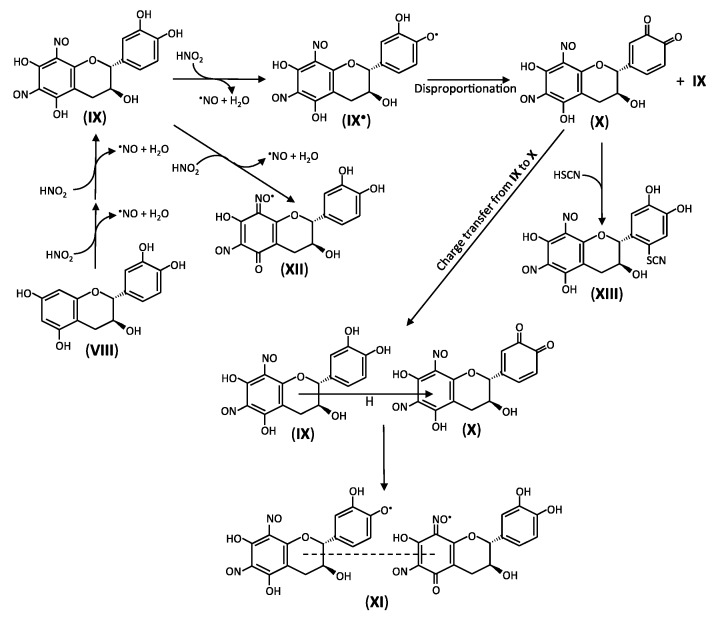
Postulated mechanism of the formation of charge-transfer complex and SCN-catechin conjugate [61,67]. (**VIII**) (+)-catechin, (**IX**) 6,8-dinitrosocatechin, (**IX^•^**) phenoxyl radical of **IX**, (**X**) quinone of **IX**, (**XI**) charge transfer complex formed from **IX** and **X**, (**XII**) nitrosyl radical of **IX**, (**XIII**) thiocyanate conjugate of **X**. (−)-Epicatechin also reacts with nitrous acid as (+)-catechin.

6,8-Dinitrosocatechin (**IX** in Figure 3) is produced during the incubation of a methanol extract of adzuki bean, which contains (+)-catechin (**VIII**), in acidified saliva [61]. Part of the formed dinitrosocatechin isomerizes to 6,8-dinitrosoepicatechin. 6,8-Dinitrosocatechin is also produced by the incubation of adzuki-meshi with nitrite, which is prepared by cooking non-glutinous rice with adzuki bean, under simulated gastric conditions [44]. Furthermore, 6,8-dinitrosoepicatechin is produced by the incubation of a methanol extract of apple fruits with acidified saliva [62] and by the decrease in the pH of masticated apple fruits to 2 [63]. The nitrosation of (−)-epicatechin accompanies the formation of dinitrosoprocyanidin B2 from procyanidin B2 contained in apple fruits [62,63], and the nitroso group is localized in the A ring of each epicatechin moiety of the procyanidin. It has been reported that (−)-epicatechin and epigallocatechin gallate are transformed into 6,8-dinitrosoepicatechin and 6,8-dinitrosoepigallocatechin gallate, respectively, by reacting with nitrous acid [64,65]. Nitrous acid-induced formation of dinitrosoprocyanidin B2 has also been reported using reagent procyanidin B2 [65]. Taking the above reports into consideration, it is expected that the dinitrosocatechins may be produced in the stomach after drinking catechin-rich green tea. As the mechanism of the nitrosation of (+)-catechin and (−)-epicatechin, the following reactions are proposed [61].

HNO_2_ + catechins → •NO + catechin radicals + H_2_O (5)

•NO + catechin radicals → mononitrosocatechins
(6)

HNO_2_ + mononitrosocatechins → •NO + mononitrosoatechin radicals (7)

•NO + mononitrosocatechin radicals → 6,8-dinitrosocatechins
(8)

The participation of the radical intermediates is postulated from the reports that semiquinone radicals of (+)-catechin participate in the formation of nitrated catechin [12]. The nitrosation, however, in the A ring of catechins and procyanidin B2 suggest that radicals, which can react with •NO, are localized in the A ring of the above compounds [66], and the nitrosation by reactions 5–8 suggest that the efficiency of •NO production by catechin/nitrous acid systems is lower than that of •NO production by quercetin and its glycoside/nitrous acid systems [51]. Quercetin and its 7-*O*-glucoside can partly suppress the dinitrosocatechin formation [43,51]. The suppression is postulated to be due to the scavenging of catechin radicals.

6,8-Dinitrosocatechin produced via reactions 5–8 can also be oxidized by nitrous acid producing the quinone (**X**) [61] (Figure 3). The formation of the quinone is effectively suppressed by quercetin and its 7-*O*-glucoside but not rutin and quercetin 4′-*O*-glucoside, suggesting that the presence of both a catechol group in the B ring and a 3-hydroxyl group in the C ring is important to suppress the oxidation of 6,8-dinitrosocatechin [43,51]. The quinone can produce the charge transfer complex (**XI**) with 6,8-dinitrosocatechin, generating nitroxyl radicals in the complexes [67]. The complexes are moderately stable; the half-life at pH 2 is about 45 min. Accompanying the increase in the pH to 7, the signals of the nitroxyl radical decrease, producing the unstable phenoxyl radicals. This result suggests that if absorbed into the body or transported to the intestine, the quinone and the charge transfer complex may be cytotoxic by reacting with cellular components and/or producing reactive oxygen species. In addition, nitroxyl radical of 6,8-dinitrosocatechin (**XII**) can also be produced by nitrous acid-dependent oxidation of 6,8-dinitrosocatechin [67]. The concentration of nitroxyl radical or charge transfer complexes is effectively decreased by ascorbic acid, producing monodehydroascorbic acid radical, and partly decreased by thiocyanic acid [67]. These results suggest that if the charge transfer complexes are produced in the stomach, they can be deactivated partly by salivary thiocyanate and by ascorbic acid when absorbed into the body. Although dinitrosocatechins can be oxidized by nitrous acid as described above, a possible function of the nitrosocatechins as inhibitor of Caco-2 cell growth has been proposed [65]. 

Ascorbic acid can reduce the quinone and the radicals to the mother compounds, while thiocyanic acid can deactivate the quinone of 6,8-dinitrosocatechin by producing stable 6′-thiocyanate-6,8-dinitrosocatechin (**XIII**) [61]. The thiocyanate conjugate does not transform into an oxathiolone derivative. A reason for the failure of its formation is that the SCN group is not adjacent to a hydroxyl group. 

### 2.4. Interactions of Floavonoids with Starch

Nitrous acid-induced oxidation of flavonoids is affected by starch. The oxidation of 100 μM quercetin becomes slower, whereas the oxidation of 100 μM kaempferol becomes faster and then slower with the increase in starch concentration from 0 to 100 mg/mL without affecting their reaction products [68]. The rates of their oxidation are related to the rates of •NO production. In addition, the oxidation rate of 20 μM vignacyanidin, which is more hydrophobic than kaempferol, increases with the increase in starch concentration from 0 to 100 mg/mL [69]. Vignacyanidin is a reddish purple cyanidin-catechin conjugate isolated from adzuki bean [70]. The above reports suggest that nitrous acid-induced oxidation of flavonoids in the stomach may be affected by starch. The effects of starch on the formation of oxathiones and thiocyanate conjugates remain to be studied.

On the other hand, it has been reported that naringin and neohesperidin mainly inhibited amylose digestion, whereas hesperidin and nobiletin inhibited both amylose and amylopectin digestion, suggesting that the inhibition was due to the formation of starch-flavonoid complexes [71]. Flavones from bamboo leaf can inhibit starch digestion by interacting with starch [72], and kaempferol, quercetin, and vignacyanidin can also inhibit starch digestion by forming the starch-flavonoid complexes [68,69]. Furthermore, starch digestion of dough produced from buckwheat flour is enhanced by the extraction of the flavonoids, supporting that the digestibility of starch can decrease by forming complexes with flavonoids [73]. If the digestion of starch-flavonoid complexes is slow, it is possible to develop foods that can decrease glycemic index using flavonoids.

## 3. Summarization of the Reactions in Nitrous Acid-Flavonoid Systems

**Figure 4:**
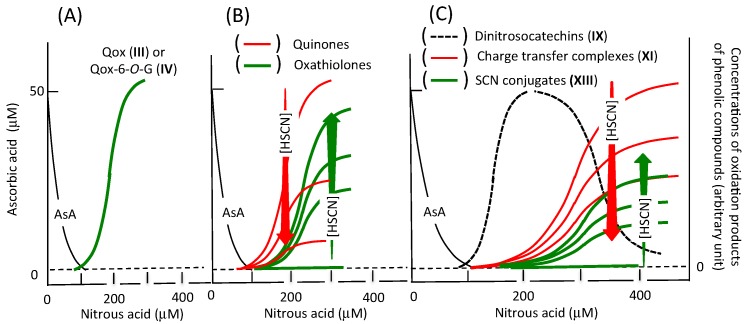
Formation of oxidation products of phenolic compounds as a function of nitrous acid concentration. Initial concentration of ascorbic acid (AsA) is postulated to be 50 μM and the concentration of nitrous acid is postulated to increase up to 400 μM. (**A**) quercetin or quercetin 7-*O*-glucoside; (**B**) caffeic acid, chlorogenic acid, or rutin; (**C**) (+)-catechin or (–)-epicatechin. Red downward and green upward arrows indicate increase in thiocyanate concentration. Its increase suppresses the formation of quinones and charge transfer complexes (red curves) and enhances the formation of oxathiolones and SCN conjugates (green curves).

Figure 4 summarizes how the above-mentioned products are formed in the presence of a defined concentration of ascorbic acid in acidic aqueous solutions, when nitrous acid concentration is increased. This figure was produced taking the published experimental data into account. The oxidation of phenolic compounds is inhibited effectively by ascorbic acid, and the oxidation can be observed after almost all of ascorbic acid has been oxidized [37,38]. The initial stable oxidation products of quercetin and its 7-*O*-glucoside are Qox (**III**) and Qox-6-*O*-G (**IV**), respectively (**A**) [43,51]. Reactive quinones formed from caffeic acid, chlorogenic acid, and rutin are transformed into the stable oxathiolone derivatives by thiocyanic acid (**B**) [33,53]. 6,8-Dinitrosocatechins formed from (+)-catechin and (–)-epicatechin are oxidized by nitrous acid to their quinones producing charge transfer complexes, and the quinones also react with thiocyanic acid producing the stable thiocyanate conjugates (**C**) [44,61,67]. Then, the scavenging of toxic quinones and/or charge-transfer complexes produced in the stomach can increase with the increase in the concentration of thiocyanic acid in saliva.

In addition, Figure 4 suggests that (i) if the gastric concentration of nitrous acid is lower than that of ascorbic acid, the oxidation of phenolic compounds does not proceed, resulting in the increase in the efficiency of the transport of phenolic compounds to the intestine, and that (ii) the reactions of phenolic compounds with nitrous acid may result in the suppression of carcinogenic nitrosoamine formation in the stomach as has been discussed previously [65,74,75]. Further studies, however, on the nitrous acid-induced oxidation of phenolic compounds in ingested foods are necessary under simulated stomach conditions to discuss the beneficial and detrimental effects of their products.
